# The Relationship Between Low-Density Lipoprotein Cholesterol and Progression of Mild Cognitive Impairment: The Influence of rs6859 in *PVRL2*

**DOI:** 10.3389/fgene.2022.823406

**Published:** 2022-02-21

**Authors:** Qianyi Xiao, Jianxiong Xi, Ruru Wang, Qianhua Zhao, Xiaoniu Liang, Wanqing Wu, Li Zheng, Qihao Guo, Zhen Hong, Hua Fu, Ding Ding

**Affiliations:** ^1^ Department of Preventive Medicine and Health Education, School of Public Health, Fudan University, Shanghai, China; ^2^ Institute of Neurology, Huashan Hospital, Fudan University, Shanghai, China; ^3^ National Clinical Research Center for Aging Diseases, Shanghai, China

**Keywords:** mild cognitive impairment, progression, Alzheimer’s disease, rs6859, low-density lipoprotein

## Abstract

**Background:** Genome-wide association studies have identified many Alzheimer’s disease (AD) genetic-risk single nucleotide polymorphisms (SNPs) and indicated the important role of the cholesterol/lipid metabolism pathway in AD pathogenesis. This study aims to investigate the effects of cholesterol and genetic risk factors on progression of mild cognitive impairment (MCI) to AD.

**Methods:** We prospectively followed 316 MCI participants aged ≥50 years with a baseline cholesterol profile and SNP genotyping data for 4.5 years on average in a sub-cohort of the Shanghai Aging Study. Total cholesterol, low-density lipoprotein cholesterol (LDL-C), and high-density lipoprotein cholesterol in serum were measured at baseline. SNP genotyping was performed using a MassARRAY system. At follow-up, consensus diagnosis of incident dementia and AD were established based on medical, neurological, and neuropsychological examinations. Cox regression models were used to assess the association of cholesterol and SNP with incident AD.

**Results:** The AG/AA genotypes of *PVRL2* rs6859 were significantly associated with increased incident AD in MCI participants, compared with GG genotype (adjusted hazard ratio [HR] 2.75, 95% confidence interval [CI] 1.32–5.76, *p* = .007, false discovery rate–adjusted *p* = .030). In *PVRL2* rs6859 AG/AA carriers, each-1 mmol/L higher level of LDL-C was significantly associated with a 48% decreased risk of AD (adjusted HR 0.52, 95%CI 0.33–0.84, *p* = .007). Consistent results were obtained when using LDL-C as the categorical variable (*P* for trend = 0.016).

**Conclusion:** The relationship between LDL-C and progression of MCI may be influenced by genetic variants.

## Introduction

Mild cognitive impairment (MCI) is an intermediate stage between normal cognition and Alzheimer’s disease (AD) (Scarabino et al., 2016). Reported annual conversion rates of MCI to AD range from 10% to 15% (Petersen et al., 1999) while the annual incidence of AD in people without dementia is between 2.1% and 3.9% (Ohara et al., 2017; Rajan et al., 2019). Several genetic and environmental risk factors have been demonstrated in the occurrence and progression of AD, such as the presence of the *Apolipoprotein E* (*APOE*) ε4 allele (Corder et al., 1993), AD-risk single nucleotide polymorphisms (SNPs) (Lambert et al., 2013), advancing age (Amieva et al., 2004), female sex (Li et al., 2016), low education attainment (Solfrizzi et al., 2004), diabetes mellitus (DM), and hypertension (Kryscio et al., 2013).

Previous epidemiological studies have explored the cholesterol–AD relationship but with inconsistent results, suggesting the cholesterol–cognition association was inconclusive. Some studies have found that a high level of cholesterol, especially low-density lipoprotein cholesterol (LDL-C), was significantly associated with an increased risk of AD (Benn et al., 2017; Schilling et al., 2017). However, insignificant and even opposing associations were concluded from other studies (Li et al., 2005; Reitz et al., 2005; Reitz et al., 2010; Zhou et al., 2018). Our previous studies based on the Shanghai Aging Study found that among older adults without vascular risk factors, TC and LDL-C were inversely associated with incident dementia, LDL-C was inversely associated with incident AD, and incremental TC and LDL-C showed significant correlation with slower annual decline of the MMSE score (Ding et al., 2021).

In recent years, a review of the AD related Genome-wide association study (GWAS) indicated that several AD risk SNP-related genes may cluster in cholesterol and lipid metabolism pathways (Van Cauwenberghe et al., 2016), such as *APOE*, clusterin (*CLU*), ATP-binding cassette transporter A7 (*ABCA7*), and poliovirus receptor–related 2 (*PVRL2*) (Ma et al., 2018; Mahley and Rall, 2000; Van Cauwenberghe et al., 2016). The Framingham Study explored a significant interaction between the genetic risk score (GRS) based on AD susceptibility loci and triglyceride level, but not cholesterol level, on the risk of AD in a cohort of European descent (Peloso et al., 2018a). However, studies that tested the association of cholesterol, AD-risk genetic variants that associated with lipid/cholesterol metabolism, and progression of MCI to AD (MCI-AD progression) were rarely seen in an older Chinese population. We hypothesize that AD-risk genetic variants that associated with lipid/cholesterol metabolism would influence the effect of cholesterol on MCI-AD progression and aim to test this hypothesis in a prospective, community-based cohort study.

## Materials and Methods

### Study Population

The Shanghai Aging Study is a longitudinal, community-based cohort study initiated in 2010 in central downtown Shanghai (Ding et al., 2014). We identified 696 individuals with MCI, aged 50 years and over, at baseline and established an MCI sub-cohort. The detailed procedures involved in an MCI diagnosis have been reported elsewhere (Ding et al., 2015).

### Ethics Committee Approval

The present study was approved by the Medical Ethics Committee of Huashan Hospital at Fudan University (No. 2009-195) and the Ethics Committee of the Department of Public Health at Fudan University (No. 2018-01-0662), Shanghai, China. All participants, or their legally acceptable representative, provided written informed consent to participate in this study.

### Data Collection at Baseline

At baseline, demographic and characteristic data were collected *via* a face-to-face questionnaire survey, including birth date, gender, height, weight, education years, Mini-Mental State Exam (MMSE) score, Center for Epidemiological Survey-Depression Scale (CES-D) score, tobacco smoking and alcohol drinking (categorized as present or never), medical histories of DM, hypertension, coronary heart diseases (CHDs), and stroke (classified as yes or no). CHDs included coronary artery disease, vascular heart disease, cardiomyopathy, heart failure, and arrhythmias. Detailed procedures of data collection have been reported elsewhere (Ding et al., 2015; Ding et al., 2016). Body mass index (BMI) was calculated as weight in kilograms divided by height in meters squared.

### Assessment of Cognitive Function at Follow-Up

Follow-up was conducted until 31 October 2016, with a median follow-up time of 4.5 years (Ding et al., 2018). Research coordinators contacted all participants with MCI and asked for a clinical interview. Those who could not be traced, refused to participate, or were deceased were defined as “lost-to-follow-up.”

At the in-person clinical interview at follow-up, participants (or the proxy) were rated on the Clinical Dementia Rating scale for cognitive complaints (Lim et al., 2007). If a person was diagnosed as having dementia or AD by neurologists at other hospitals, the time and hospital names were recorded. Participants were measured on the Lawton and Brody Activity of Daily Living (ADL) scale, and functionally intact of physical self-maintenance and instrumental activities of daily living were considered for whose ADL score >16 (Lawton and Brody, 1969). Participants with new onset of stroke were examined for their motor responses and reflexes. The onset and subtype of stroke were queried from medical records, and the results of prior computed tomography/magnetic resonance imaging images were recorded. The cognitive function of participants was tested using the same neuropsychological battery that was used at baseline (Ding et al., 2015). The test battery covered the domains of global cognition, executive function, spatial construction function, memory, language, and attention, including 1) MMSE; 2) Conflicting Instructions Task (Go/No Go Task); 3) Stick Test; 4) Modified Common Objects Sorting Test; 5) Auditory Verbal Learning Test; 6) Modified Fuld Object Memory Evaluation; 7) Trail-making tests A and B; and 8) Renminbi (Chinese currency) test. Neuropsychological tests were administered by study psychometrists according to the education level of each participant. Normative data and a detailed description of these tests have been reported elsewhere (Ding et al., 2015). All tests were conducted in Chinese within a 90-min timeframe.

Neurologists and neuropsychologists in our study group reviewed clinical and neuropsychological data and reached a consensus diagnosis of incident AD using the Diagnostic and Statistical Manual of Mental Disorders-IV and the National Institute of Neurological and Communicative Disorders and Stroke and the Alzheimer’s Disease and Related Disorders Association criteria (American Psychiatric Association, 1994). Diagnostic procedures and criteria were the same as those at baseline.

### Serological Testing

A baseline blood sample was collected from each participant by research nurses in the morning after 12 h of overnight fasting. Serum cholesterol profiles were measured using a Hitachi 7600 fully automatic biochemical analyzer in the central laboratory of Huashan Hospital. Total cholesterol (TC) was measured using an oxidase method, and LDL-C and high-density lipoprotein cholesterol (HDL-C) were measured using a direct method.

### SNP Selection, Genotyping, and Quality Control

For SNP selection, first, we selected the SNPs which were identified in GWAS with an AD-risk association that exceeded the threshold of a genome-wide significance level (*p* < 5 × 10^−8^) in European and Asian populations before June 2019; if multiple SNPs are in strong linkage disequilibrium (LD) and met the above criterion, defined by pairwise *r* ^*2*^ < 0.2 estimated from the HapMap CHB (Han Chinese in Beijing, China) population, the most commonly cited SNP was selected. Next, the SNP-related genes that were reported to be involved in cholesterol/lipid metabolism pathways in publications were included in this study. Finally, only four SNPs were selected and their information is listed in Table 1. Of these SNPs, although *PVRL2* rs6859 is located on chromosome 19, the same as for *APOE*, there is no strong linkage disequilibrium between *PVRL2* rs6859 and *APOE* (treating ε3 and ε2 as the same allele and ε4 as another allele) with *r* ^*2*^ = 0.13 reported in our previous study (Xiao et al., 2015), and *r* ^*2*^ = 0.28 in this study.

**TABLE 1:** Selected AD GWAS-SNPs in cholesterol/lipid metabolism–related genes evaluated in this study.

Region	SNP ID [ref.]	Closest gene	Position	Allele^a^	Risk allele^b^	MAF (CHB)^c^	MAF (observed)^d^	HWE	Gene pathway [ref.]
8p21.1	rs11136000 (Seshadri, et al., 2010)	*CLU*	27520436	C/T	C	0.199	0.222	1	Cholesterol and lipid metabolism (Van Cauwenberghe et al., 2016)
8p21.1	rs569214 (Antunezet al., 2011)	*CLU*	27543709	T/G	G	0.456	0.476	0.650	Cholesterol and lipid metabolism (Van Cauwenberghe et al., 2016)
19p13.3	rs4147929 (Lambertet al., 2013)	*ABCA7*	1063444	G/A	A	0.282	0.302	0.062	Lipid metabolism and immune response (Van Cauwenberghe et al., 2016)
19q13.32	rs6859 (Abrahamet al., 2008)	*PVRL2*	50073874	G/A	A	0.320	0.353	0.081	Response to plasma cholesterol lowering (Takei et al., 2009)

SNP, single nucleotide polymorphism; GWAS, genome-wide association study; HWE, Hardy-Weinberg equilibrium; CHB, han chinese in beijing, China; MAF, minor allele frequency; ref., reference; CLU, clusterin; ABCA7, ATP- binding cassette transporter A7; PVRL2, poliovirus receptor‐ related 2.

a Major/Minor.

b Risk allele reported in European population.

c MAF in Chinese Han population in Hapmap database.

d MAF observed in present study.

Genomic DNA was extracted from peripheral blood samples at baseline using a QIAamp DNA Blood Mini kit (QIAGEN GmbH, Hilden, Germany). Genotyping of selected SNPs was performed on a MassARRAY system (iPLEX; Sequenom Inc., San Diego, CA, United States) by using an Agena Biosciences (San Diego, CA, United States) iPLEX Gold Genotyping reagent. Four duplicate test samples and four water samples (PCR negative controls) that the technician was blinded to were included in each 384-well plate to monitor genotyping accuracy. The average concordance rate was 100% among these duplicate samples. All assays were conducted by technicians blinded to participant status. SNPs with missing SNPs >1, minor allele frequency (MAF) < 0.01, or *p* < .001 in a Hardy–Weinberg Equilibrium (HWE) test were removed.

*APOE* genotype was measured using a TaqMan SNP method (Smirnov et al., 2009). Because there are only three participants carrying *APOE* ε4/ε4 in our study, the presence of at least one ε4 allele was defined as *APOE* ε4 positive in the following analysis.

### Statistical Analysis

A one-way ANOVA was used for a comparison of continuous variables, and a chi-square test was used for categorical variables. LDL-C values were divided into three levels according to tertile: low (<2.86 mmol/L), medium (2.86–3.67 mmol/L), and high (>3.67 mmol/L). Participants with MCI who had not converted to AD at the last follow-up visit were regarded as censored. The effect of SNPs on MCI-AD progression was assessed using a Cox regression model, adjusted for age and gender in model 1 and additionally adjusted for *APOE* ε4 status and each genetic variant in model 2. When we examined the effects of baseline cholesterol concentrations on MCI-AD progression, age, gender, *APOE* ε4 status, and education years were adjusted in Cox regression model 1; BMI, DM, hypertension, CHDs, stroke, smoking, drinking, and lipid-lowering medication were additionally adjusted in model 2. Covariates selected in analyses were based on the criteria of “the variable that is related to AD risk, but not in the causal pathway between cholesterol and AD risk,” and also were referred to relevant literatures (Schilling et al., 2017; Peloso et al., 2018b; Chung et al., 2018). Cumulative incidence graphs of MCI-AD conversion between LDL-C categories were drawn using a Cox regression model, adjusting for age, gender, *APOE* ε4 status, education years, and lipid-lowering medication, and stratified into different genetic-risk groups. The hazard ratio (HR) and 95% confidence interval (CI) were used as measures to assess the risk effect.

Statistical analyses were performed using PLINK 1.07 software and SPSS 21.0 software. All tests were two-sided and *p* < .05 was considered as statistically significant. The false discovery rate (FDR), as proposed by Benjamini and Hochberg, was calculated using R software for multiple comparison (Benjamini et al., 2001).

## Results

### Key Demographic and Clinical Information of Participants

**FIGURE 1:**
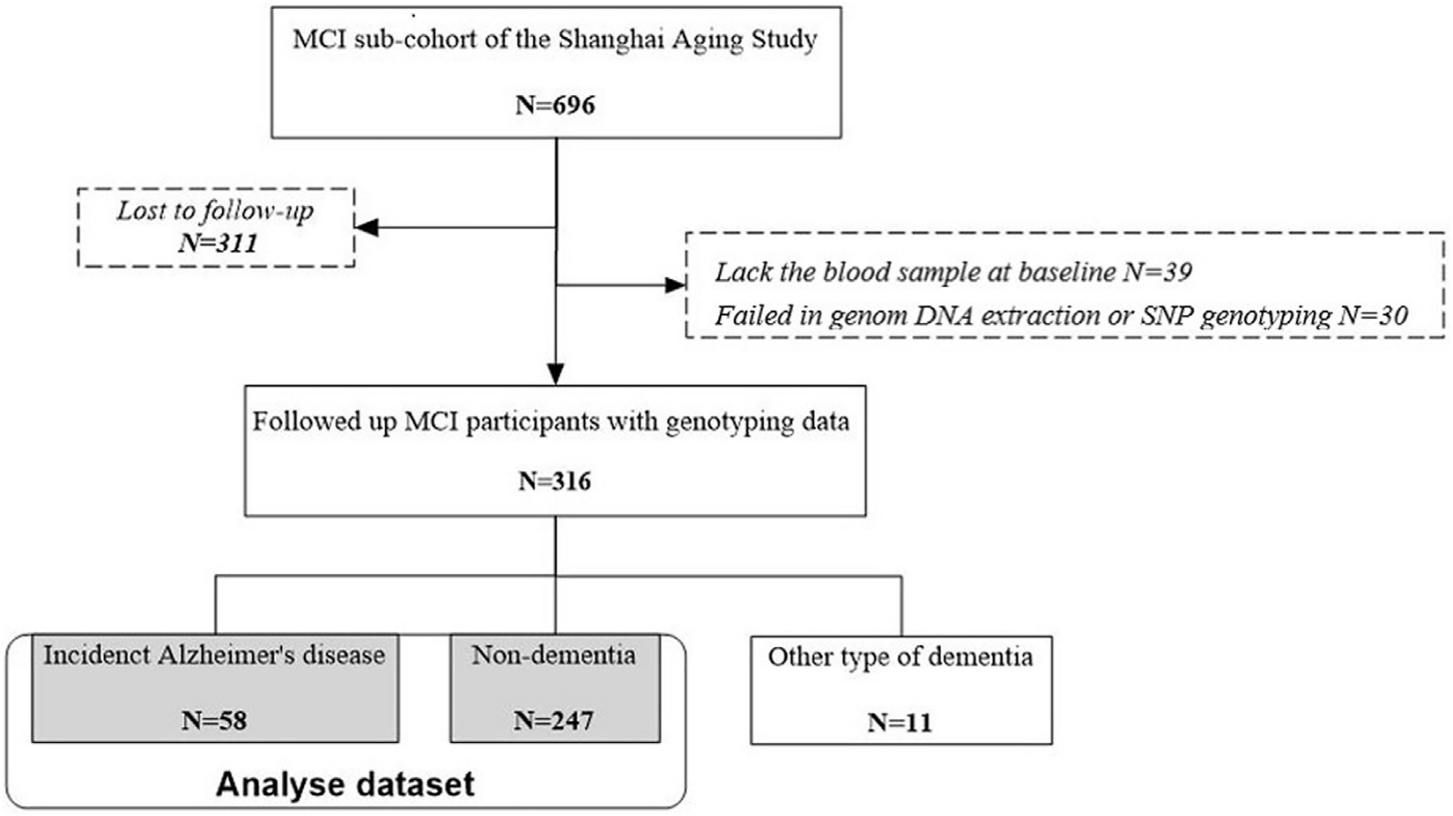
Flow chart of participant recruitment for the present study. MCI, mild cognitive impairment.

Of 696 MCI participants at baseline, 311 were lost-to-follow-up, 39 lacked baseline blood samples, and 30 were failed in genomic DNA extraction or SNP genotyping. After an average of 4.5 years [standard deviation (SD) 1.3] of follow-up, we diagnosed 58 with incident AD, 11 with other types of dementia, and 247 as non-demented. We therefore included 305 participants with incident AD and non-dementia in our dataset for analysis (Figure 1). The excluded MCI participants were slightly but statistically significantly older (74.7 vs. 72.7, *p* = .003), with lower MMSE score (*p* < .001), lower education years (*p* = .016), and lower BMI level (*p* = .035), than the included MCI participants in the analysis. There was no significant difference between these two groups in sex, *APOE* ε4, CES-D score, medical history, cholesterol concentrations at baseline, and smoking and drinking status (Supplementary Table S1).

As shown in Table 2, the mean age of the included 305 MCI participants at baseline was 72.7 (SD 8.0) years, the mean education attainment was 10.5 (SD 4.5) years, and the mean MMSE score was 27.1 (SD 2.5). It was found that 20.9% of MCI participants were *APOE* ε4 positive. Participants with a medium LDL-C level were older than those with low or high LDL-C (*p* = .012). Participants with high LDL-C were more likely to be women (*p* = .023) and to have higher TC (*p* < .001) and HDL-C (*p* = .003) levels at baseline. Of the three groups with different LDL-C levels, there was no significant difference in *APOE* ε4, education year, MMSE score, CES-D score, lipid-lowering medication, BMI, medical history, and smoking and drinking status at baseline, and AD onset during follow-up.

**TABLE 2:** Characteristics of MCI participants.

Characteristic	Total^a^	LDL-C (mmol/L)^b^			*P*
		Low (<2.86)	Medium (2.86-3.67)	High (>3.67)	
Participants, n (%)	305 (100)	101	102	102	
Age at baseline (years)	72.7 (8.0)	71.6 (7.6)	74.6 (8.4)	71.7 (7.9)	**.012**
Women, n (%)	170 (55.7)	47 (46.5)	56 (54.9)	67 (65.7)	**.023**
*APOE* ε4 positive, n (%)^c^	60 (20.9)	14 (14.9)	21 (21.2)	25 (26.6)	.142
Education years (years)	10.5 (4.5)	10.8 (4.2)	10.4 (4.7)	10.3 (4.6)	.658
MMSE score at baseline	27.1 (2.5)	27.0 (2.4)	27.1 (2.7)	27.1 (2.4)	.933
CES-D score at baseline	10.0 (9.1)	11.1 (9.6)	9.1 (8.5)	9.9 (9.0)	.293
Lipid-lowering medication, n (%)	26 (8.5)	9 (9.8)	9 (8.8)	8 (7.8)	.955
Vascular risk factors at baseline					
BMI (kg/m^2^)	24.4 (3.9)	24.3 (3.7)	24.8 (4.5)	24.2 (3.4)	.441
Diabetes mellitus, n (%)	53 (17.4)	20 (20.0)	21 (20.6)	12 (11.8)	.179
Hypertension, n (%)	168 (55.1)	57 (56.4)	60 (58.8)	51 (50.0)	.424
Stroke, n (%)	47 (15.4)	15 (14.9)	17 (16.7)	15 (14.7)	.911
CHDs, n (%)	41 (13.5)	13 (12.9)	18 (17.8)	10 (9.8)	.241
Smoking, n (%)	34 (11.2)	15 (15.0)	9 (8.8)	10 (9.8)	.327
Drinking, n (%)	29 (9.5)	7 (6.9)	14 (13.7)	8 (7.8)	.200
Baseline cholesterol concentrations					
TC (mmol/L)	5.1 (1.4)	4.4 (0.7)	5.1 (0.8)	6.1 (1.0)	**< .001**
HDL-C (mmol/L)	1.3 (0.4)	1.2 (0.4)	1.3 (0.4)	1.4 (0.5)	**.003**
LDL-C (mmol/L)	3.2 (1.1)	2.4 (0.7)	3.2 (0.4)	4.2 (0.7)	**< .001**
Incident AD in follow-up, n (%)	58 (19.0)	22 (21.8)	20 (19.6)	16 (15.7)	.533

Values are shown as mean (standard deviation) or number (percent). Baseline cholesterol concentrations were performed as median (interquartile range). Bold values indicate statistically significant.

AD, Alzheimer’s disease; *APOE*, Apolipoprotein E; BMI, body mass index; CES-D, center for epidemiological survey, Depression Scale; CHDs, coronary heart diseases; HDL-C, high-density lipoprotein cholesterol; LDL-C, low-density lipoprotein cholesterol; MCI, mild cognitive impairment; MMSE, Mini-Mental State Exam; TC, total cholesterol.

a Participants with incident Alzheimer’s disease and non-dementia at follow-up were included in our analyze dataset.

b Baseline LDL-C, concentration was divided into three tertiles [low: < 2.86 mmol/L, medium: 2.86-3.67 mmol/L and high: > 3.67 mmol/L].

### Genotypes and MCI-AD Progression

All four SNPs passed the quality control standards. The associations of genetic variants with MCI-AD progression were analyzed using a Cox regression model adjusted for age and gender in model 1 and additionally adjusted for *APOE* ε4 status and other three SNPs in model 2. The *PVRL2* rs6859 AG, AA, and AG/AA genotypes were significantly associated with increased MCI-AD progression, compared with GG genotype (AG vs. GG, HR (95%CI) = 2.53 (1.14–5.58), *p* = .022, *FDR (P)* = .040; AA vs. GG*,* HR (95%CI) = 3.12 (1.30–7.83), *p* = .011, *FDR (P)* = .030; AG/AA vs. GG, HR (95%CI) = 2.75 (1.32–5.76), *p* = .007, *FDR (P)* = .030, Table 3, model 2). In *APOE* ε4 non-carriers, *PVRL2* rs6859 still had a significant association with MCI-AD progression adjusting for age and gender (AG/AA vs. GG, HR (95% CI) = 2.23 (1.07–4.65), *p =* .032); however, *PVRL2* rs6859 and *APOE* had no interactive effect on MCI-AD progression (*P*_inter rs6859×*APOE*_ = 0.567) (Supplementary Table S2)*.* In addition, in our study, *APOE* ε4 was found to be associated with increased risk of incident AD in univariate analysis (*p* = .045) but not in multivariate analysis adjusting for age and gender (*p* = .077), compared to the *APOE* ε4 non-carriers (Supplementary Table S3). These findings suggested that AG/AA in *PVRL2* rs6859 was a genetic risk factor of conversion from MCI to AD.

**TABLE 3:** Multivariate Cox regression analysis of genotypes of SNPs on risk of MCI-AD progression.

Genotype	Number of patients	Number of events	Model 1			Model 2		
			HR (95%CI)	*P*	*FDR (P)*	HR (95%CI)	*P*	*FDR (P)*
*CLU* rs11136000								
CC	185	40	Ref			Ref		
TC	105	15	0.60 (0.33-1.09)	.094	.078	0.66 (0.33-1.32)	.244	.183
TT	15	3	0.99 (0.30-3.21)	.982	.437	0.67 (0.14-3.13)	.606	.335
TC/TT			0.64 (0.37-1.12)	.121	.092	0.69 (0.37-1.27)	.230	.178
*CLU* rs569214								
TT	80	18	Ref			Ref		
GT	155	22	0.64 (0.34-1.20)	.167	.117	0.67 (0.31-1.43)	.300	.205
GG	65	17	1.40 (0.72-2.71)	.325	.205	1.24 (0.58-2.65)	.574	.323
GT/GG			0.85 (0.48-1.48)	.557	.306	0.92 (0.47-1.79)	.804	.400
Missing data	5							
*ABCA7* rs4147929								
GG	155	26	Ref			Ref		
AG	114	26	1.97 (1.14-3.43)	**.016**	**.021**	1.80 (0.94-3.43)	.074	.081
AA	35	6	1.20 (0.49-2.92)	.693	.354	1.22 (0.46-3.23)	.692	.365
AG/AA			1.74 (1.04-2.95)	**.035**	**.037**	1.73 (0.96-3.11)	.068	.078
Missing data	1							
*PVRL2* rs6859								
GG	131	13	Ref			Ref		
AG	120	32	2.85 (1.48-5.51)	.002	**.005**	2.53 (1.14-5.58)	**.022**	**.040**
AA	46	12	3.14 (1.42-6.93)	.005	**.008**	3.12 (1.30-7.83)	**.011**	**.030**
AG/AA			2.93 (1.65-5.50)	**.001**	**.005**	2.75 (1.32-5.76)	**.007**	**.030**
Missing data	8							

Model 1 was adjusted for age, gender; Model 2 was adjusted for age, gender, *APOE*, and other three SNPs. MCI, mild cognitive impairment; AD, Alzheimer’s disease; SNP, single nucleotide polymorphism; CI, confidence interval. Bold values indicate statistically significant.

a The total number of patients were different in each genetic variant group due to different missing data of genotyping failing for each variant.

### Cholesterol and MCI-AD Progression

**FIGURE 2:**
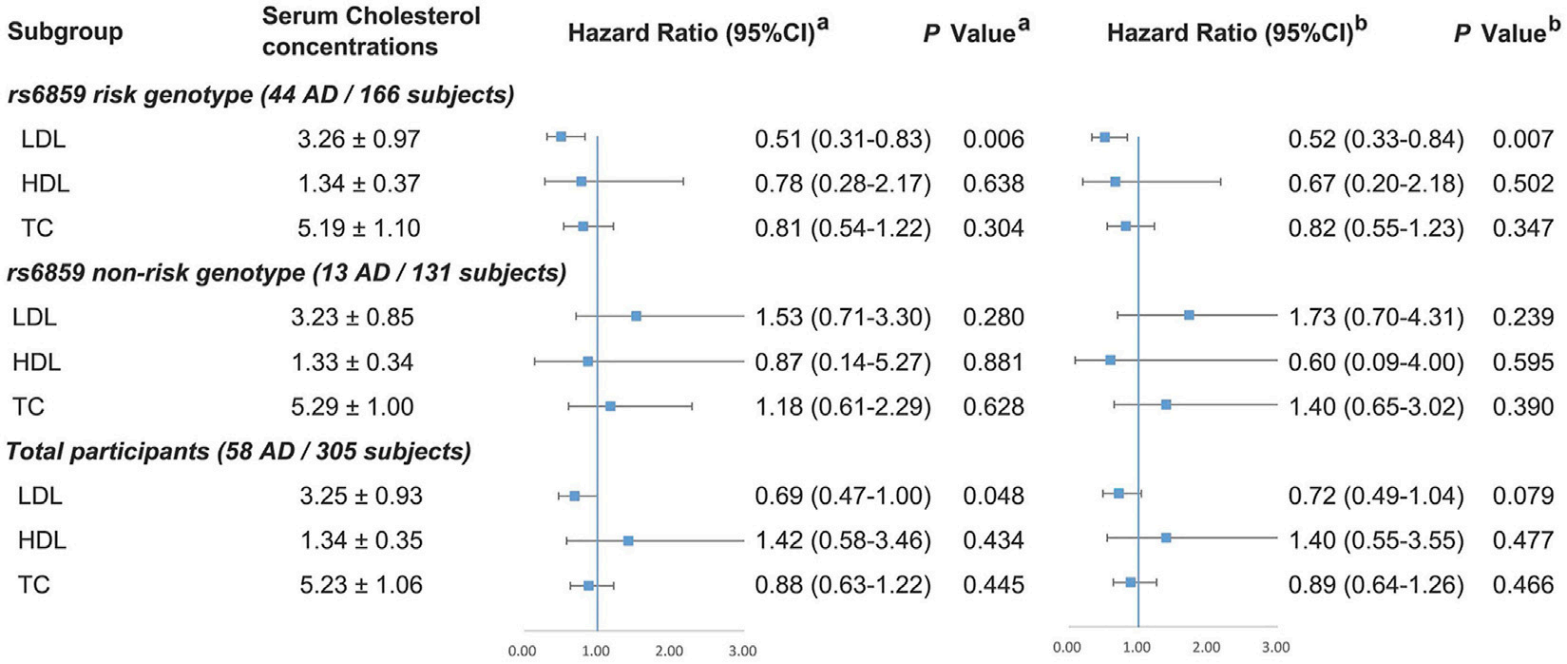
Association between cholesterol and MCI-AD progression among total participants and in subgroups stratified by *PVRL2* rs6859 genotypes. *PVRL2* rs6859 AG/AA was defined as risk genotype and GG was non-risk genotype. ^**a**^ Model 1 was adjusted for age, gender, *APOE* ε4 status, and education years. ^**b**^ Model 2 was additionally adjusted for vascular risk factors (body mass index, diabetes mellitus, hypertension, coronary heart disease, stroke, smoking, and drinking) and lipid-lowering medication. *APOE*, Apolipoprotein E; TC, total cholesterol; HDL-C, high-density lipoprotein cholesterol; LDL-C, low-density lipoprotein cholesterol. Note: There were eight missing data of genotyping failing for *PVRL2* rs6859.

The distributions of cholesterol in different *PVRL2* rs6859 genotypes or *APOE* ε4 status were examined before further analysis, and no significantly different distributions were found (Supplementary Table S4). The association of cholesterol with MCI-AD progression is detailed in Figure 2. Among *PVRL2* rs6859 AG/AA carriers, each 1 mmol/L higher level of LDL-C was significantly associated with 48% decreased risk of AD in model 2 (HR (95%CI) = 0.52 (0.33–0.84), *p =* .007, model 2). However, no significant associations between HDL-C, LDL-C, TC concentration, and MCI-AD progression were found in *PVRL2* rs6859 GG carriers. Among total samples, LDL-C (per mmol/L) level was inversely associated with incident AD in Cox regression model 1 (HR = 0.69, 95%CI = 0.47–1.00, *p* = .048), but the association was no longer significant in model 2 (*p* = .079). Meanwhile, we examined the association of cholesterol with MCI-AD progression in subgroups stratified by *APOE* ε4 status and found that LDL-C was also inversely associated with incident AD in *APOE* ε4 carriers in Cox regression (HR = 0.43, 95%CI = 0.19–0.97, *p* = .043; Supplementary Figure S1, model 2).

**FIGURE 3:**
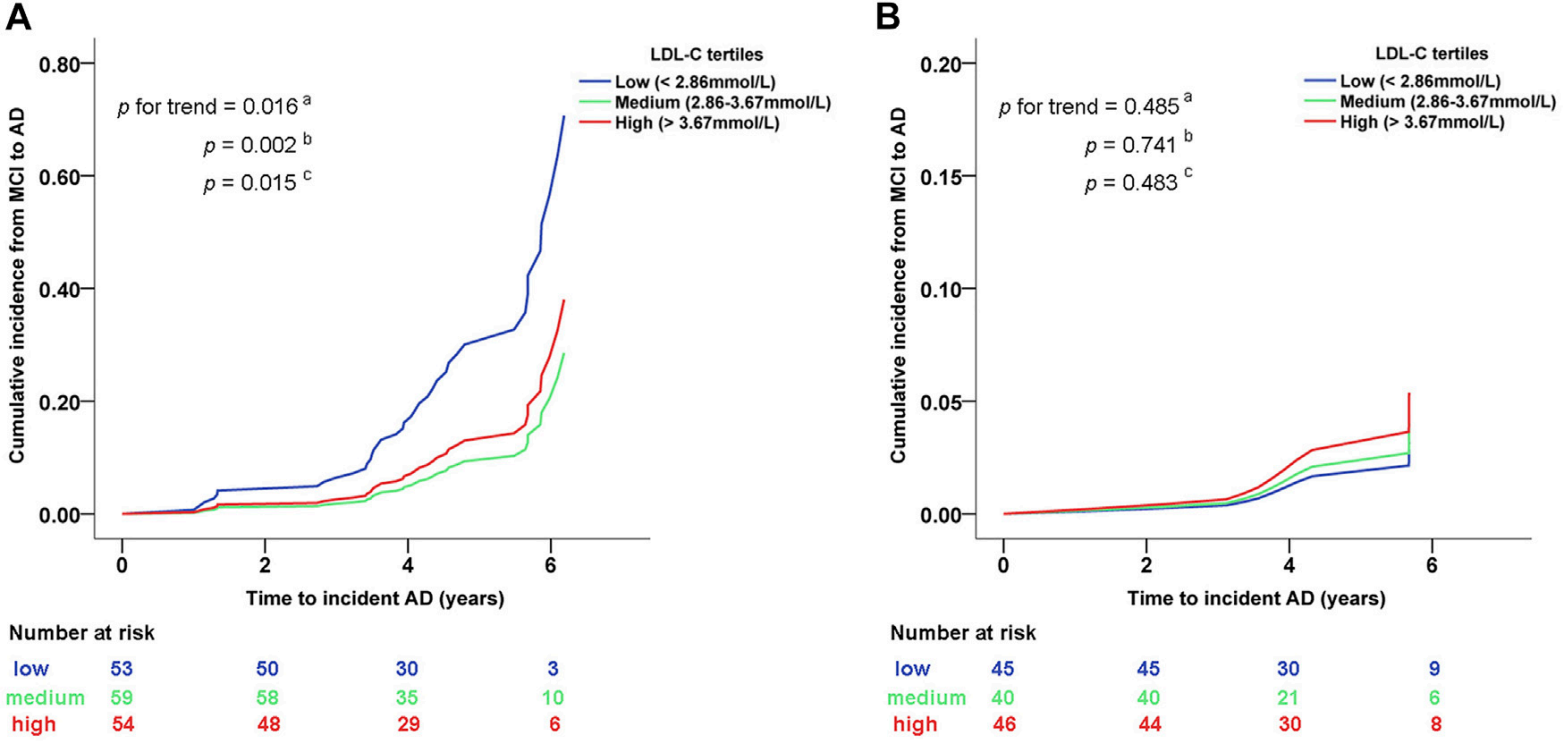
Cumulative conversion rate from MCI to AD between different LDL-C category levels in subgroups stratified by *PVRL2* rs6859 genotypes. **(A)** *PVRL2* rs6859 AG/AA genotype subgroup. **(B)** *PVRL2* rs6859 GG genotype subgroup. Cumulative incidence graphs are based on age, gender, *APOE* ε4 status, education years, and lipid-lowering medication adjusted Cox models (from which indicated *p* values were extracted). ^**a**^ Trend *p* of cumulative incidence across the three groups of participants with different levels of LDL-C. ^**b**^ medium vs. low LDL-C. ^**c**^ high vs. low LDL-C. AD, Alzheimer’s disease; MCI, mild cognitive impairment; LDL-C, low-density lipoprotein cholesterol.

We further divided the LDL-C concentration into categorical scales by tertile. As shown in Figure 3, among MCI participants with *PVRL2* rs6859 AG/AA genotype, those maintaining medium/high LDL-C (2.86-3.67 mmol/L/> 3.67 mmol/L) had a significantly decreased cumulative risk of AD compared with those with low LDL-C (<2.86 mmol/L) (*P* for trend = .016, *P*_*medium vs. low*_ *=* .002*, P*_*high vs. low*_ *=* .015; Figure 3A). No significant association between LDL-C and cumulative risk of AD was found in *PVRL2* rs6859 GG carriers (Figure 3B). These findings indicated that among MCI with *PVRL2* rs6859 AG/AA genotypes, LDL-C was a risk factor of progression from MCI to AD.

## Discussion

The present community-based prospective study suggests an inverse association between LDL-C at baseline and risk of incident AD in MCI participants with an AD genetic risk factor of *PVLR2* rs6859 AG/AA. However, no significant association was found in those with *PVRL2* rs6859 GG genotype or total MCI participants. Our results suggest that genetic factors might influence the relationship between LDL-C and risk of incident AD, providing a possible explanation for inconsistent LDL-C-cognition association from previous population-based study (Li et al., 2005; Reitz et al., 2005; Reitz et al., 2010; Benn et al., 2017; Schilling et al., 2017; Zhou et al., 2018) and suggesting that among individuals with *PVRL2* rs6859 AG/AA, aggressively lowering LDL-C might not be beneficial to prevent cognition impairment.

The mechanism by which *PVRL2* rs6859 influences the relationship between LDL-C and risk of AD remains elusive. *PVRL2* rs6859 was previously reported to be associated with AD risk (Hollingworth et al., 2011) and poor cognitive performance (Cruz-Sanabria et al., 2018) and also involved in cholesterol and lipid metabolism. Based on the HaploReg database, (http://pubs.broadinstitute.org/mammals/haploreg/haploreg.php), the rs6859 locates in the 3′UTR region of *PVRL2* gene, and the 3′UTR can control gene expression by affecting the localization, stability, and translation of mRNAs (Decker and Parker, 1995). The Genotype-Tissue Expression (GTEx) database (http://www.gtexportal.org/) also shows that the different genotypes of rs6859 were significantly correlated with the expression level of *PVRL2* gene in many tissues, including brain hippocampus, whole blood, artery-aorta, artery-tibial tissues, and artery-coronary tissue (Supplementary Figure S2). Specifically, the association of rs6859 genotypes with *PVRL2* gene expression remained significant in whole blood, artery-aorta, and artery-tibial tissues even after multiple test correction. Compared to those with GG genotypes, the *PVRL2* gene expression level in patients with AG and AA genotype was significantly decreased (Supplementary Figure S3). Furthermore, *PVRL2* knockout mice were reported to have less atherosclerosis (Rossignoli, Shang, Gladh, Moessinger, and Foroughi et al., 2017). Importantly, our previous study based on the Shanghai Aging Study found that among older adults without vascular risk factors, LDL-C was inversely associated with incident AD (Ding et al., 2021). All these findings above might provide a reasonable explanation for the results of the present study, that is, older adults with rs6859 AA/AG genotypes might experience decreased expression of *PVRL2* in the body and then have less cardiovascular risk. Therefore, among older adults with rs6859 AA/AG genotypes, LDL-C is inversely associated with incident AD.

Importantly, our results showed that *PVRL2* rs6859 was significantly associated with MCI-AD progression. Even in *APOE* ε4 non-carriers, *PVRL2* rs6859 still had a significant association with MCI-AD progression (Supplementary Table S2). This finding is particularly important in the assessment of MCI-AD progression. *APOE* has been irrefutably recognized as the major genetic risk factor for late-onset AD (Chartier-Harlin et al., 1994). Although Asian populations have a lower *APOE* ε4 frequency (6.9%) (Hu et al., 2011) than Europeans (10–15% in the south to 40–50% in the north) (Ewbank, 2004), the prevalence of cognitive impairment in the Chinese population (Hilal et al., 2013) is still in a similar range to the prevalence reported in Caucasians (Alexander et al., 2015). Therefore, other genetic variants may also contribute to the pathological process of AD and influence the susceptibility of AD in the Chinese population. The evidence for the frequency *APOE* ε4 in MCI is mostly unexplored. Only a few studies have been conducted with, as yet, small sample sizes (ranging from 28 to 583 participants) and with the *APOE* ε4 frequency ranging from 7.5% to 39.6% in China, France, Spain/Catalonia, Switzerland, Belgium, and Cuba, as summarized by Tsolak (Tsolaki et al., 2018). MCI participants in our study also showed low prevalence of *APOE* ε4 carriers (20.9%, 60/290, Table 2) but a high prevalence of *PVRL2* rs6859 AG/AA carriers (55.9%, 166/297, Table 3). Even among non-*APOE* ε4 carriers, 46.2% (102/221) participants carried the *PVRL2* rs6859 AG/AA genotype (Supplementary Table S2). Therefore, *PVRL2* rs6859 may supplement *APOE* for better assessing the AD genetic risk in the Chinese population.

Differing from previous prospective cohort studies that examined the cholesterol effect on AD progression from cognitive normal (Ancelin et al., 2013; Reitz et al., 2005; Schilling et al., 2017), we prospectively followed up a cohort of MCI participants and assessed the risk of incident AD. MCI is an intermediate transition stage between cognitive normal and dementia; thus, our study on MCI-AD progression may provide the evidence for the second AD prevention and have the advantage of saving observation time and improving efficiency. Another advantage is that we provided a precise evaluation of AD genetic susceptibility when examining the cholesterol effect on MCI-AD progression, that is, the genetic polymorphisms selected in this study were both identified in an AD GWAS and reported to be involved in cholesterol/lipid metabolism.

*PVRL2*, also known as *NECTIN2*, is a cell membrane protein located in the LDL-C GWAS locus *APOE*. The relationship between *PVRL2* and LDL-C level is complicated. Takeiet al. reported that *PVRL2* is a cholesterol-responsive gene expressed in many organs, including the brain (Takei et al., 2009). *PVRL2* was also found to be markedly downregulated in response to plasma cholesterol lowering in atherosclerosis-prone mice with a humanlike plasma cholesterol profile (Rossignoli et al., 2017). Recently, a Mendelian randomization study reported a negative causal relationship between *PVRL2* expression LDL-C uptake in hepatic cells, but a positive causal relationship between *PVRL2* expression in liver and LDL-C levels in plasma (van der Graaf et al., 2020). In our study, the LDL-C levels neither differ in different *PVRL2* rs6859 genotypes nor in different *APOE* ε4 status (Supplementary Table S4).

Several longitudinal studies have examined the effects of lipids on AD. However, the results are conflicting (Li et al., 2005; Reitz et al., 2005; Reitz et al., 2010; Ancelin et al., 2013; Proitsi et al., 2014; Benn et al., 2017; Schilling et al., 2017) due to ethics differences, different stages when lipids were measured, onset age, and follow-up duration (Benn et al., 2017; Reitz et al., 2010; Schilling et al., 2017). The Framingham Heart Study (*n* = 3040) examined the effects of mid-life lipid levels on AD based on AD-risk genetic variants and found that the effect of triglycerides on AD varies according to the sortilin-related receptor 1 (*SORL1) SNP* rs11218343, but no interaction occurred between AD-risk SNPs and the LDL-C level on the risk of AD. However, this study was conducted in older European adults, aged 65 years and over, and with a 10-year follow-up from cognitive normal to AD (Peloso et al., 2018a). Previous Mendelian randomization studies have explored the causal nature of the relationship between lipids and AD using lipid metabolism–related genetic variants (Benn et al., 2017; Proitsi et al., 2014). One Mendelian randomization study, including participants from the Copenhagen City Heart Study (*n* = 11,201) and Copenhagen General Population Study (*n* = 99,993), reported that low LDL-C, due to genes responsible for LDL-C metabolism and biosynthesis, was associated with decreased AD risk in the European population with a median follow-up time of 8.2 years (Benn et al., 2017). However, another Mendelian randomization study, combining British Birth Cohort (*n* = 9398), Institute of Psychiatry Plus group (*n* = 663), and Alzheimer’s Disease Neuroimaging Initiative data (*n* = 517), suggested that lipid-related genetic variants were not causally associated with late-onset AD risk through changes in the LDL-C level in the European population (Proitsi et al., 2014). Our findings offered evidence that lipid metabolism–related SNPs may affect the role of cholesterol in MCI-AD progression in older Chinese people.

In analysis of the cumulative conversion rate from MCI to AD between different LDL-C category levels in high-risk groups stratified by *PVRL2* rs6859 genotypes (Table 2), patients with medium LDL-C concentration have the lowest risk of conversion from MCI to AD. LDL-C is a well-established causal risk factor for cardiovascular disease (Silverman et al., 2016), which in turn is recognized as a risk factor of AD. Therefore, the reason for the non–dose-dependent manner in the relationship between LDL-C concentration and the conversion rate may be that excessively low level of LDL-C may partially neutralize the beneficial role of LDL-C for MCI-AD progression through contributing to the risk of cardiovascular disease (de la Torre, 2004; Kelleher and Soiza, 2013).

Limitations should be acknowledged. First, although our study involved a population-based cohort, its sample size was relatively small, which may skew our results to be statistically insignificant. For example, *APOE* was not observed as a significant risk factor on MCI-AD progression in our study in the multivariate Cox regression model. Second, the mean follow-up time of MCI participants was relatively short. However, as an intermediate state between cognitive normal and AD, MCI has a relative higher conversion rate to AD than a cognitive normal status (18% in our study) within a short follow-up time. Therefore, this average 4.5-year follow-up study with 305 participants was able to achieve a sufficient statistical power in analysis. Third, the Shanghai Aging Study was composed of people with Han Chinese backgrounds, and therefore, these results may not be generalizable to other ethnic populations. Finally, because the excluded participants were older and have a worse baseline cognitive performance than the included participants, the association between LDL-C and the risk of MCI progressing to AD in our study may be underestimated due to the lower follow up rate among very old residents. This should be taken into account when generalizing our results to the general population.

In summary, results from this study suggest that the effect of LDL-C on MCI-AD progression may influenced by genetic variants. Among older people with genetic risk of *PVRL2* rs6859 AG/GG, maintaining a moderate and slightly high LDL-C level might be beneficial in preventing AD onset in this subgroup. Larger studies in other ethnic populations with longer follow-up are needed to validate our findings and to explore the potential pathological mechanisms.

## Data Availability

Data in the current study are available from the corresponding author on reasonable request and with permission of Huashan Hospital. Additionally, the genotype data of participants can be shared with a signed data confidentiality contract to ensure that the use of the data will meet the requirements of China's Ministry of Science and Technology (MOST) and the data will not be leaked to a third party.
