# Supplementary material for: The Relationship Between Low-Density Lipoprotein Cholesterol and Progression of Mild Cognitive Impairment: The Influence of rs6859 in PVRL2
Source: Front Genet. 2022 Feb 21;13:823406. doi: 10.3389/fgene.2022.823406 (PMC8901437; doi:10.3389/fgene.2022.823406)
Supplement: Supplementary file 1 [file DataSheet1.docx]

**1. Supplementary Figures and Tables**

**1.1 Supplementary Figures**

**
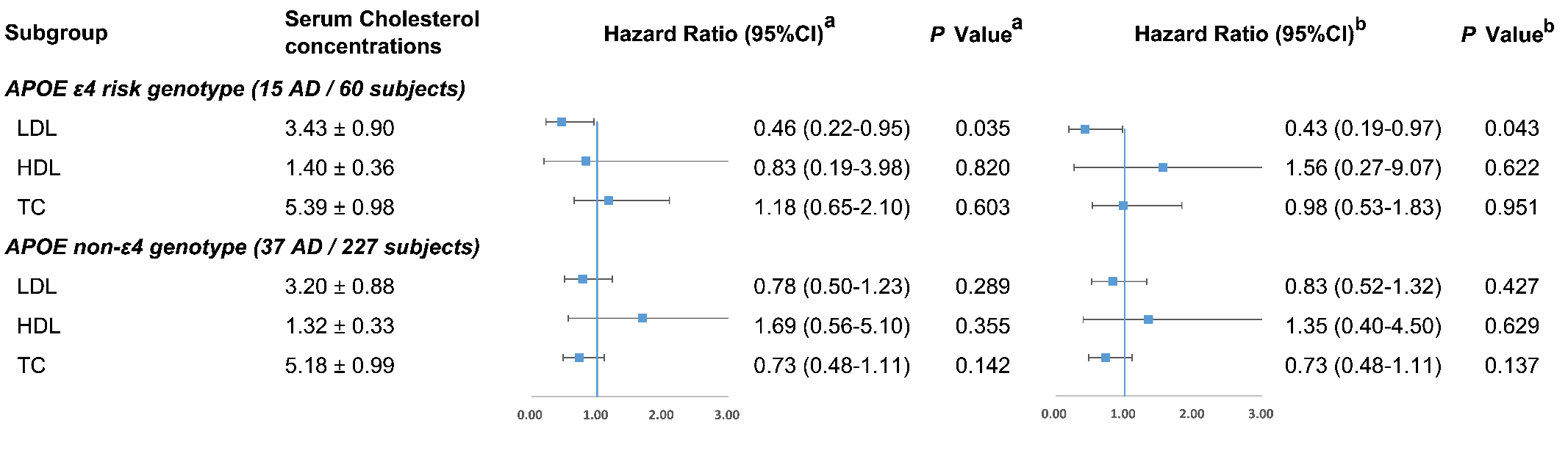
**

**Supplementary Figure 1.** The association between cholesterol and AD risk in subgroups stratified by *APOE* ε4 status.

**^a^** Model 1 was adjusted for age, gender, education years. **^b^** Model 2 was additionally adjusted for vascular risk factors (body mass index, diabetes mellitus, hypertension, coronary heart disease, stroke, smoking and drinking) and lipid-lowering medication. *APOE*, Apolipoprotein E; TC, total cholesterol; HDL-C, high-density lipoprotein cholesterol; LDL-C, low-density lipoprotein cholesterol. Note: There are 287 participants with the APOE genotype data.


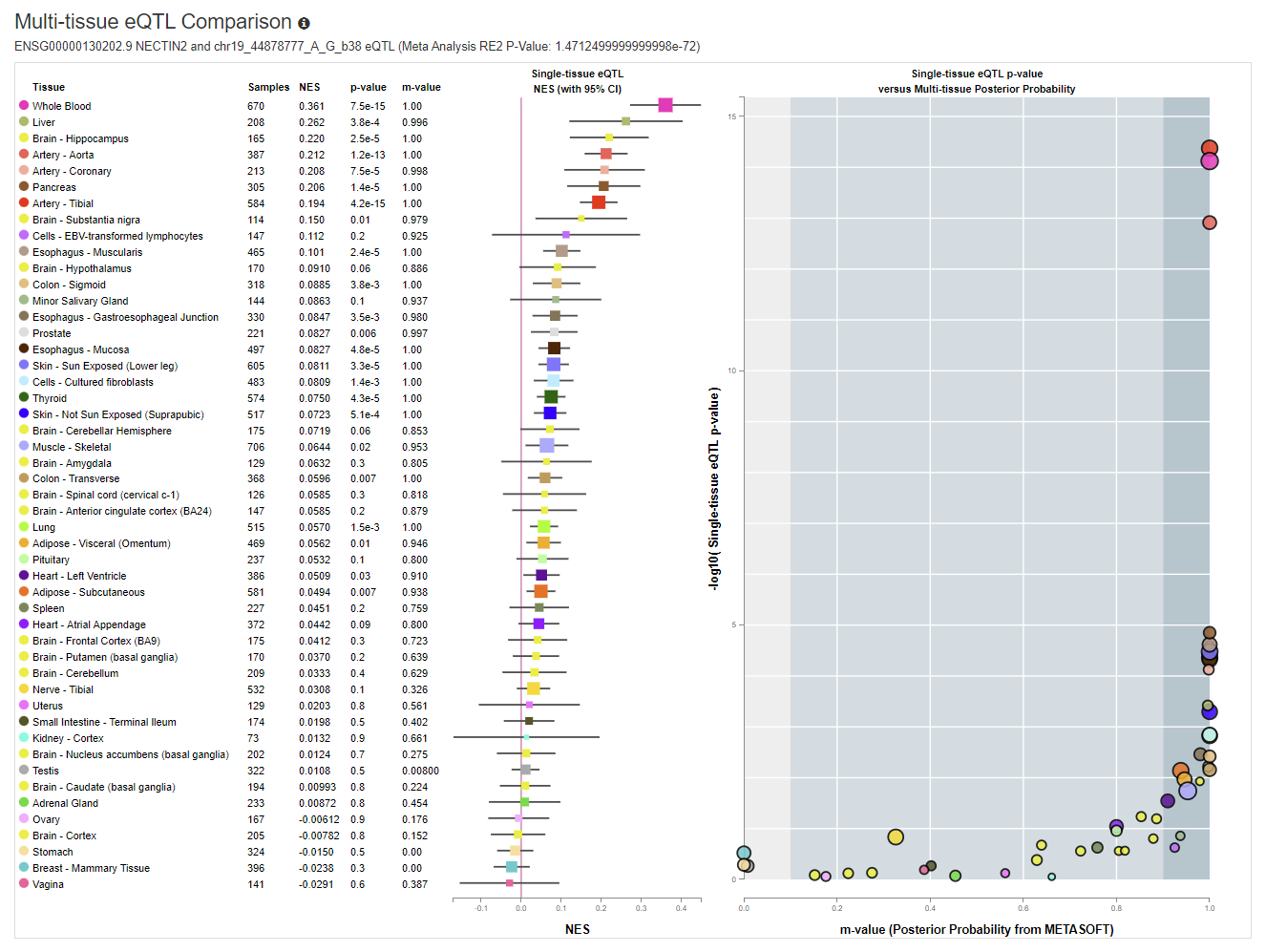


**Supplementary Figure 2** Multi-tissue eQTL (expression Quantitative Trait Locus) analyses for *PVRL2* (*NECTIN2* is an alias of *PVRL2)* expression and rs6859based on Genotype-Tissue Expression (GTEx) database ([*http://www.gtexportal.org/*](http://www.gtexportal.org/)).

**
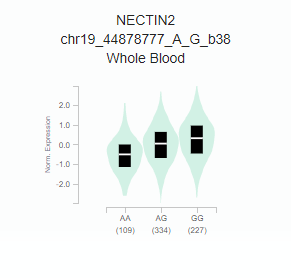

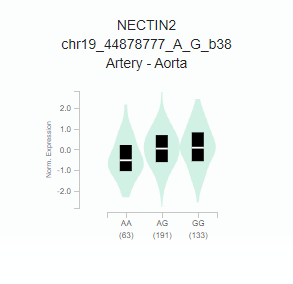

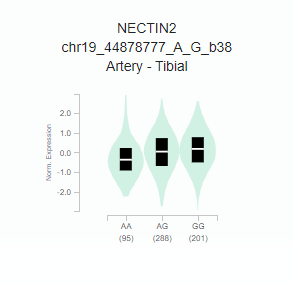
**

**Supplementary Figure 3** Expression level of *PVRL2* (*NECTIN2* is an alias of *PVRL2*) gene in patients with different genotypes of rs6859 in whole blood, artery-aorta and artery-tibial tissues based on Genotype-Tissue Expression (GTEx) database ([*http://www.gtexportal.org/*](http://www.gtexportal.org/)).

**1.2 Supplementary Tables**

**Supplementary Table 1.** Baseline characteristics of the MCI cohort

|  | All MCI participants (n=696) | | *P* value |
| --- | --- | --- | --- |
|  | Excluded MCI  (N = 391) | Included MCI  (N = 305) |  |
| Age at baseline, (years) | 74.7 ± 9.9 | 72.7 ± 8.0 | **0.003** |
| Women, n (%) | 224 (57.3) | 170 (55.7) | 0.168 |
| *APOE* ε4 positive, n (%) | 63 (19.3) | 60 (20.9) | 0.257 |
| MMSE at baseline | 26.1 ± 3.0 | 27.1 ± 2.5 | **<0.001** |
| Education years, (years) | 9.6 ± 4.9 | 10.5 ± 4.5 | **0.016** |
| CESD at baseline | 10.1 ± 8.8 | 10.0 ± 9.1 | 0.913 |
| BMI, (kg/m^2^) | 23.8 ± 4.1 | 24.4 ± 3.9 | **0.035** |
| Diabetes mellitus, n (%) | 65 (16.8) | 53 (17.4) | 0.840 |
| Hypertension, n (%) | 240 (62.0) | 168 (55.1) | 0.066 |
| Stroke, n (%) | 70 (18.0) | 47 (15.4) | 0.369 |
| CHDs, n (%) | 72 (18.5) | 41 (13.5) | 0.076 |
| Smoking, n (%) | 51 (13.1) | 34 (11.2) | 0.450 |
| Drinking, n (%) | 30 (7.7) | 29 (9.5) | 0.391 |
| TC, (mmol/L) | 5.34 ± 0.92 | 5.14 ± 1.42 | 0.211 |
| HDL-c, (mmol/L) | 1.35 ± 0.36 | 1.34 ± 0.40 | 0.768 |
| LDL-c, (mmol/L) | 3.30 ± 0.86 | 3.22 ± 1.08 | 0.637 |

Note: MCI, mild cognitive impairment; AD, Alzheimer’s disease; APOE, Apolipoprotein E; MMSE, Mini-Mental State Exam; BMI, body mass index; CES-D, Center for Epidemiological Survey; Depression Scale; CHDs, coronary heart diseases; TC, total cholesterol; HDL-C, high-density lipoprotein cholesterol; LDL-C, low-density lipoprotein cholesterol.

**Supplementary Table 2.** Multivariate Cox regression analysis of *PVRL2* rs6859 and MCI-AD progression in subgroups stratified by *APOE* genotypes

| Genotype | No. of patients ^a^ | No. of events | Cox regression model | | |
| --- | --- | --- | --- | --- | --- |
|  |  |  | HR | 95%CI | *p* Value |
| *APOE* ε4 |  |  |  |  |  |
| rs6859-GG | 6 | 0 | - | - | - |
| rs6859-AG/AA | 52 | 14 | - | - | - |
| *APOE* non-ε4 |  |  |  |  |  |
| rs6859-GG | 119 | 12 | Ref. | - | - |
| rs6859-AG/AA | 102 | 25 | 2.23 | 1.07-4.65 | **0.032** |
| *P*_inter rs6859×_*_APOE_* = 0.567 | | | | | |

Note: Multivariate Cox regression model was adjusted for age and gender.

MCI, mild cognitive impairment; AD, Alzheimer’s disease; SNP, single nucleotide polymorphism; CI, confidence interval; *APOE*, Apolipoprotein E.

^a^ There are 279 participants with both *APOE* and *PVRL2* rs6859 genotype data, in which there are 6 rs6859-GG and 52 rs6859-AG/AA carriers in the *APOE* ε4 positive group for a total of 58 patients, and 119 rs6859-GG and 102 rs6859-AG/AA carriers in the *APOE* non-ε4 group for a total of 221 patients.

**Supplementary Table 3.** Univariate and Multivariate Cox regression analysis of *APOE* on risk of MCI-AD progression.

| Genotype | No. of patients ^a^ | No. of events | Univariate analysis | | | Multivariate analysis | | |
| --- | --- | --- | --- | --- | --- | --- | --- | --- |
|  |  |  | HR | 95%CI | *P* Value | HR | 95%CI | *P* Value |
| *APOE* | | |  |  |  |  |  |  |
| Non-ε4 | 227 | 37 | Ref. |  |  | Ref. |  |  |
| At least one ε4 ^b^ | 60 | 15 | 1.85 | 1.01-3.39 | 0.045 | 1.73 | 0.94-3.19 | 0.077 |

Note: Multivariate Cox regression model was used for calculating hazard ratio (95% confidence interval) and P value, adjusting for age and gender.

^a^ There are 287 participants with the *APOE* genotype data, in which there are 60 *APOE* ε4 carriers and 227 *APOE* non-ε4 carriers.

^b^ One ε4 carriers: n=57; Two ε4 carriers: n=3.

**Supplementary Table 4.** Association of *PVRL2* rs6859 and *APOE* genotypes with cholesterol levels

| Cholesterol levels | rs6859 GG genotypes | | rs6859 AG/AA genotypes | | *P* | *APOE* non-ε4 | | *APOE* ε4 | | |  |
| --- | --- | --- | --- | --- | --- | --- | --- | --- | --- | --- | --- |
|  | Mean ± Std | Median (IQR) | Mean ± Std | Median (IQR) |  | Mean ± Std | Median (IQR) | Mean ± Std | Median (IQR) | | *P* |
| LDL | 3.23 ± 0.85 | 3.15 (1.11) | 3.27 ± 0.97 | 3.10 (1.12) | 0.889 | 3.20 ± 0.88 | 3.15 (1.11) | 3.43 ± 0.90 | 3.40 (1.22) | 0.054 | |
| HDL | 1.33 ± 0.34 | 1.30 (0.40) | 1.34 ± 0.37 | 1.30 (0.48) | 0.795 | 1.32 ± 0.33 | 1.30 (0.40) | 1.40 ± 0.36 | 1.40 (0.47) | 0.159 | |
| TC | 5.29 ± 1.00 | 5.09 (1.36) | 5.19 ± 1.10 | 5.10 (1.34) | 0.364 | 5.18 ± 0.99 | 5.09 (1.36) | 5.39 ± 0.98 | 5.34 (1.24) | 0.122 | |

Note: *APOE*, Apolipoprotein E; Std, standard deviation; IQR, interquartile range; TC, total cholesterol; HDL-C, high-density lipoprotein cholesterol; LDL-C, low-density lipoprotein cholesterol.
